# A predictive nomogram for short‐term outcomes of myasthenia gravis patients treated with low‐dose rituximab

**DOI:** 10.1111/cns.14761

**Published:** 2024-05-13

**Authors:** Yufan Zhou, Rongjing Guo, Xingyu Xia, Sisi Jing, Jun Lu, Zhe Ruan, Sushan Luo, Xiao Huan, Chongbo Zhao, Ting Chang, Jianying Xi

**Affiliations:** ^1^ Department of Neurology, Huashan Hospital Fudan University Shanghai China; ^2^ Huashan Rare Disease Center, Huashan Hospital Fudan University Shanghai China; ^3^ National Center for Neurological Diseases Shanghai China; ^4^ Department of Neurology, Tangdu Hospital The Fourth Military Medical University Xi'an China; ^5^ Department of Neurology, Banan Hospital Chongqing Medical University Chongqing China

**Keywords:** myasthenia gravis, nomogram, predictor, prognosis, rituximab

## Abstract

**Background:**

This study aims to establish and validate a predictive nomogram for the short‐term clinical outcomes of myasthenia gravis (MG) patients treated with low‐dose rituximab.

**Methods:**

We retrospectively reviewed 108 patients who received rituximab of 600 mg every 6 months in Huashan Hospital and Tangdu Hospital. Of them, 76 patients from Huashan Hospital were included in the derivation cohort to develop the predictive nomogram, which was externally validated using 32 patients from Tangdu Hospital. The clinical response is defined as a ≥ 3 points decrease in QMG score within 6 months. Both clinical and genetic characteristics were included to screen predictors via multivariate logistic regression. Discrimination and calibration were measured by the area under the receiver operating characteristic curve (AUC‐ROC) and Hosmer–Lemeshow test, respectively.

**Results:**

Disease duration (OR = 0.987, *p* = 0.032), positive anti‐muscle‐specific tyrosine kinase antibodies (OR = 19.8, *p* = 0.007), and genotypes in *FCGR2A* rs1801274 (AG: OR = 0.131, *p* = 0.024;GG:OR = 0.037, *p* = 0.010) were independently associated with clinical response of post‐rituximab patients. The nomogram identified MG patients with clinical response with an AUC‐ROC (95% CI) of 0.875 (0.798–0.952) in the derivation cohort and 0.741(0.501–0.982) in the validation cohort. Hosmer–Lemeshow test showed a good calibration (derivation: Chi‐square = 3.181, *p* = 0.923; validation: Chi‐square = 8.098, *p* = 0.424).

**Conclusions:**

The nomogram achieved an optimal prediction of short‐term outcomes in patients treated with low‐dose rituximab.

## Graphical abstract

Our study developed and validated the clinical response prediction nomogram for myasthenia gravis patients treated with low‐dose rituximab. The nomogram can effectively identify patients with clinical response, help make treatment‐related decisions, alleviate the financial burden on patients, and rationalize the allocation of healthcare resources.

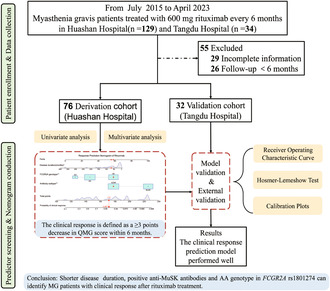

## 1 INTRODUCTION

Myasthenia gravis (MG) is an autoimmune disorder characterized by autoantibodies against neuromuscular junctions, including anti‐acetylcholine receptor (AChR) and anti‐muscle‐specific tyrosine kinase (MuSK) antibodies. Approximately 10%–15% of MG patients with anti‐AChR antibody‐positive (AChR‐MG) and most anti‐MuSK antibody‐positive MG (MuSK‐MG) patients do not respond to standard treatments, such as glucocorticoids and immunosuppressants (IS) like azathioprine, tacrolimus, or mycophenolate mofetil. Moreover, some patients experience adverse reactions to these therapies.^1^, ^2^, ^3^, ^4^, ^5^ As a result, novel biological targeting agents, such as rituximab (RTX), are increasingly being used in the treatment of MG.

RTX is a monoclonal IgG1 antibody that targets the B lymphocyte membrane protein CD20. There are still controversies regarding the target population and optimal regimen for MG treatment. Our previous studies have shown that low‐dose RTX (500–600 mg, every 6 months) is effective in treating AChR‐MG and MuSK‐MG patients.^6^, ^7^ However, the response to treatment varied among individuals, and five of 12 AChR‐MG patients showed less than 3 points reduction in the quantitative myasthenia gravis (QMG) score 6 months after the first RTX infusion.^8^

Up to now, predictors for treatment response to RTX in MG have not been investigated. In rheumatoid arthritis (RA),^9^ systemic vasculitis,^10^ neuromyelitis optic spectrum disorder (NMOSD),^11^ and systemic lupus erythematosus(SLE),^12^ both clinical parameters (such as disease severity, age at onset, age at RTX start, and gender) and biomarkers (including the titer of antibodies, the frequency of memory B cells, single‐nucleotide polymorphism(SNP) in *BAFF*, *FCGR3A*, *FCGR2A*, and *IL‐12/21*) have been shown to be related to treatment response. Since there is wide heterogenicity in the treatment efficacy among different patients, it is essential to identify predictors related to treatment response in MG.

In this study, we developed and validated a nomogram that predicts the clinical response of RTX in AChR‐MG and MuSK‐MG patients based on their baseline clinical characteristics and SNPs.

## 2 METHODS

### 2.1 Study population

This retrospective observational study included MG patients from the registry database of Huashan Hospital, Fudan University, and Tangdu Hospital, the Fourth Military Medical University, from July 2015 to April 2023. Of 1828 generalized MG patients registered at Huashan Hospital, 7.06% (129) have received rituximab treatment; in the database of 451 patients with generalized MG from Tangdu Hospital, 7.54% (34) were treated with rituximab. Inclusion criteria were as follows: (1) age ≥16 years old; (2) anti‐MuSK or anti‐AChR antibody positive; (3) QMG scores ≥3; (4) treated with 600 mg rituximab; and (5) duration from rituximab exposure to last visit was at least 6 months. Patients with incomplete clinical data at baseline were excluded. Eligible patients from Huashan Hospital with integrated data were included in the derivation cohort and patients from Tangdu Hospital were included in the validation cohort. Written informed consent was granted by each patient and the study was approved by the Institutional Review Board of Huashan Hospital, Fudan University, and Tangdu Hospital, the Fourth Military Medical University.

### 2.2 Evaluation and collection

Demographic variables in this study include gender, age at onset, and age at first RTX treatment. Clinical features before the first RTX treatment, include the comorbidities of other autoimmune diseases, disease duration, Myasthenia Gravis Foundation of America (MGFA) classification, history of crisis, thymoma concurrence, history of thymectomy, plasma exchange (PE), intravenous immunoglobulin (IVIg), IS administration, response to traditional immunotherapies, and daily dosage of prednisone, MG‐related activities of daily living (MG‐ADL) score, and related sub‐scores (bulbar, respiratory, ocular, and limb score), as well as QMG score and related sub‐scores (extraocular muscle, bulbar muscle, gross motor, and axial motor score). Disease duration is defined as the period from the onset to the first exposure to rituximab. Refractory MG, defined as MGFA postintervention status (MGFA‐PIS) unchanged or worse after steroids and at least one other nonsteroid immunosuppressant.

We divided the total QMG score into five sub‐scores: (1) Extraocular muscle score, which includes diplopia, ptosis, and facial muscles; (2) Bulbar muscle score, which includes dysarthria, and cough while swallowing water; (3) Respiratory score, which includes vital capacity, % predicted; (4) Gross motor score, which includes arms outstretched, hands grip, and legs outstretched; and (5) Axial motor score, which includes head lifted.

The MG‐ADL score was divided into four sub‐scores: (1) Ocular score: diplopia and ptosis; (2) Bulbar score: talking, chewing, and swallowing activities; (3) Respiratory score: the activity of breathing; (4) Limb score: the ability to brush teeth or comb hair, and arise from a chair.

### 2.3 Genotyping

Genomic DNA was extracted from peripheral blood according to the manufacturer's instructions. *FCGR3A* (rs396991, A > C), *FCGR2A* (rs1801274, A > G), *TNFSF13B* (rs9514828, C > T), and *TNFSF13B* (rs3759467, T > C) gene polymorphisms were analyzed by real‐time polymerase chain reaction (PCR) using TaqMan genotyping assays with probes (Applied Biosystems). Genotyping methodology was previously described.^13^, ^14^ The assay ID used for *FCGR3A* (rs396991) is C_25815666_10, for *FCGR2A* (rs1801274) is C_9077561_20, for *TNFSF13B* (rs9514828) is C_27497010_10, and for *TNFSF13B* (rs3759467) is C_29641742_10.

### 2.4 Outcome assessment

The clinical response is defined as a ≥ 3 points decrease in QMG score within 6 months.

### 2.5 Statistical analysis

All continuous variables have undergone the Shapiro–Wilk test for normality. Continuous variables that followed a normal distribution are reported as the mean ± standard deviation. Non‐normally distributed data are presented as the median (interquartile range, IQR). Categorical variables are expressed as frequencies (percentages). The comparison between cohorts was performed using chi‐squared or Fisher's exact test for categorical variables and the Student's *t*‐test or Mann–Whitney *U* test for continuous variables. Data analysis was carried out using IBM SPSS version 25.0 (SPSS Inc., Chicago, IL, USA) and R version 4.2.2 (R Foundation for Statistical Computing, Vienna, Austria). All diagrams were generated in R version 4.2.2 (R Foundation for Statistical Computing, Vienna, Austria). Statistical significance was defined as a two‐tailed *p* < 0.05.

### 2.6 Model derivation

The model was derived through the following steps: The significance of each variable in the derivation cohort was analyzed using univariate logistic regression analysis. The variance inflation factors (VIFs) were generated to examine individual predictors for potential contributions to multicollinearity. Variables that showed statistical (*p* < 0.05) and clinical significance in the univariate analysis were included in the backward multivariate logistic regression model to select independent predictors of clinical response (*p* < 0.05) and develop the nomogram.

### 2.7 Model validation

The discrimination performance of the nomogram was measured using the area under the receiver operating characteristic curve (AUC‐ROC) in both the derivation and validation cohorts, with 95% confidence intervals (95% CI) provided. Calibration was evaluated using the Hosmer–Lemeshow goodness‐of‐fit test and calibration plots. A two‐tailed *p* < 0.05 was considered significant. Model validation was conducted in two steps. First, internal validation was performed using a bootstrap resampling process to provide an unbiased estimate of model performance. Second, external validation was conducted by assessing the prediction accuracy of the clinical response nomogram on the validation cohort through the computation of AUC‐ROC and calibration plots.

## 3 RESULTS

### 3.1 Clinical characteristics

**FIGURE 1:**
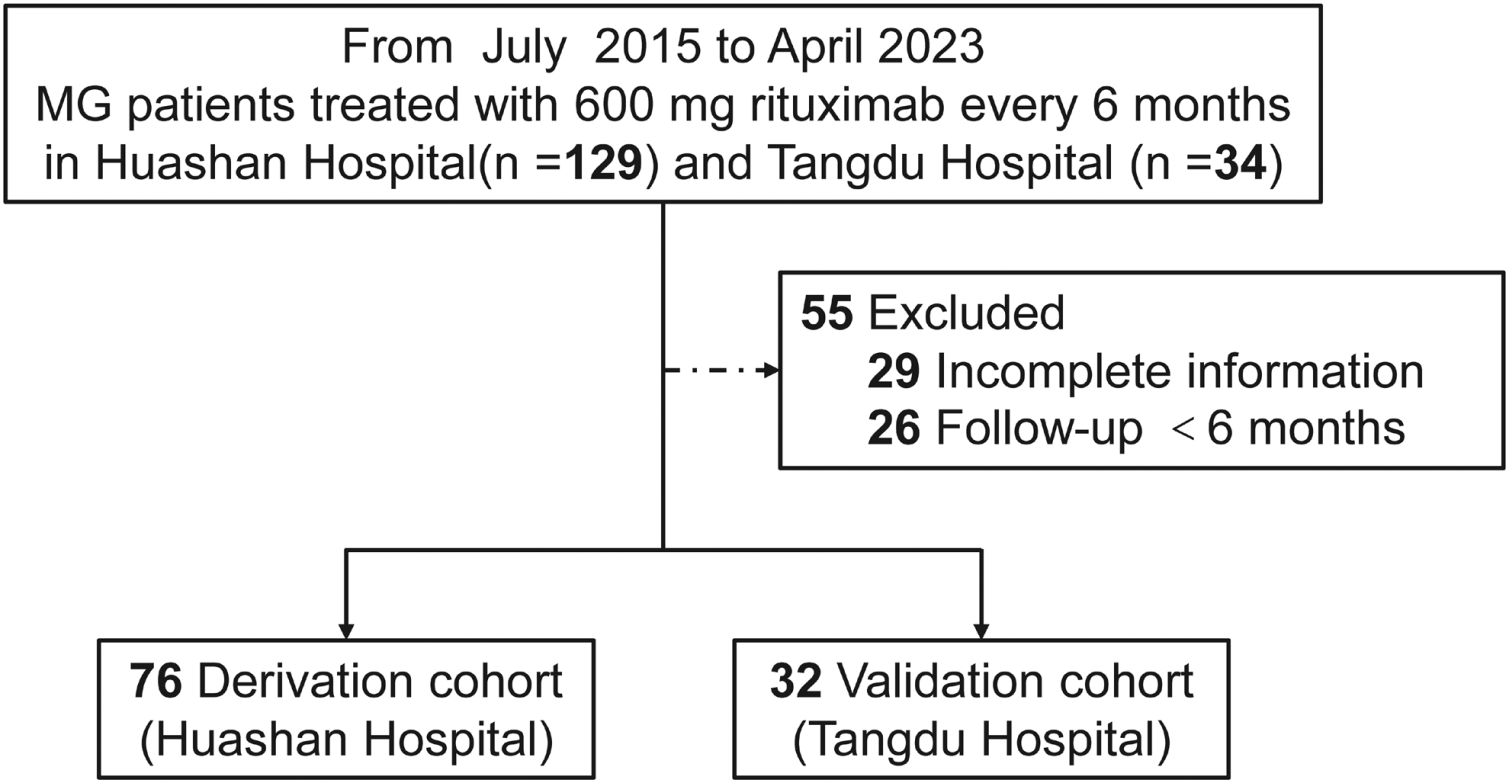
Flowchart of patient screening.

A total of 163 MG patients who were treated with 600 mg rituximab every 6 months were initially registered in two tertiary referral centers. Following the inclusion and exclusion flowchart, we finally enrolled 108 MG patients in the baseline registry. Of them, 26 AChR‐MG and 6 MuSK‐MG patients from Tangdu Hospital were enrolled for external validation (Figure 1).

Clinical and demographic profiles as well as SNPs, of the derivation and external validation cohorts are outlined and compared in Table 1. The frequency of patients achieving clinical response was similar for the derivation (76%) and external validation cohorts (88%), whereas there were some differences between the two cohorts regarding age at onset, age at the first RTX treatment, disease duration, antibody subtype, MGFA classification, comorbid autoimmune diseases, history of PE, IVIg, and thymectomy, ADL‐bulbar score, ADL‐limb score, and QMG score.

**TABLE 1:** The baseline demographic and clinical characteristics of the derivation and external validation cohort.

Variables	Derivation cohort (*n* = 76)	Validation cohort (*n* = 32)	*p* value
Gender, *n* (%)			0.122
Male	14 (18)	11 (34)	
Female	62 (82)	21 (66)	
Age at onset (years), Mean ± SD	36.47 ± 14.86	46.16 ± 17.78	*0.009*
Age at first RTX treatment (years), Mean ± SD	40.57 ± 14.45	48.47 ± 17.32	*0.028*
Disease duration (months), Median (Q1, Q3)	28.5 (13, 73)	14.5 (5.25, 36)	*0.004*
Antibody subtype, *n* (%)			*0.02*
AChR	42 (55)	26 (81)	
MuSK	34 (45)	6 (19)	
MGFA classification before first RTX, *n* (%)			*0.044*
I	2 (3)	5 (16)	
II	27 (36)	14 (44)	
III	31 (41)	11 (34)	
IV	15 (20)	2 (6)	
V	1 (1)	0 (0)	
History of crisis, *n* (%)	24 (32)	11 (34)	0.953
Comorbid autoimmune diseases, *n* (%)	15 (20)	0 (0)	*0.005*
Thymoma, *n* (%)	16 (21)	3 (9)	0.239
Thymectomy, *n* (%)	21 (28)	3 (9)	0.067
History of PE, *n* (%)	26 (34)	0 (0)	*<0.001*
History of IVIg, *n* (%)	45 (59)	11 (34)	*0.032*
Prednisone dosage, mg/d, Median (Q1, Q3)	30 (25, 40)	25 (20, 40)	0.18
Immunosuppressant administration, *n* (%)	52 (68)	18 (56)	0.323
AZA, n (%)	24 (32)	14 (44)	0.323
FK506, *n* (%)	40 (53)	3 (9)	*<0.001*
MMF, *n* (%)	4 (5)	5 (16)	0.121
MTX, *n* (%)	1 (1)	0 (0)	1
CTX, *n* (%)	9 (12)	0 (0)	0.055
Cyclosporin, *n* (%)	1 (1)	3 (9)	0.077
Refractory patients, *n* (%)	52 (68)	18 (56)	0.323
MG‐ADL, Median (Q1, Q3)	7 (5, 9)	6 (4, 7.25)	0.177
ADL‐bulbar, Median (Q1, Q3)	3 (2.4)	2 (0.25, 3)	*0.036*
ADL‐respiratory, Median (Q1, Q3)	0 (0.1)	0 (0.1)	0.472
ADL‐limb, Median (Q1, Q3)	1 (0, 2.75)	0 (0.2)	*0.025*
ADL‐ocular, Median (Q1, Q3)	2 (0.3)	2.5 (0.3)	0.923
QMG, Mean ± SD	14.47 ± 5.30	10 ± 5.75	*<0.001*
QMG‐extraocular, Median (Q1, Q3)	3 (2.5)	3 (1.25, 4)	0.085
QMG‐bulbar, Median (Q1, Q3)	2 (0.3)	1 (0.2)	*0.036*
QMG‐respiratory, Median (Q1, Q3)	1 (0,1.75)	0 (0.1)	*0.034*
QMG‐gross motor, Median (Q1, Q3)	6 (4, 10)	4 (1.75, 7.25)	*0.048*
QMG‐axial motor, Median (Q1, Q3)	2 (1.2)	1 (0.1)	*<0.001*
FCGR3A, *n* (%)			0.863
AA	37 (49)	16 (50)	
AC	31 (41)	14 (44)	
CC	8 (11)	2 (6)	
FCGR2A, *n* (%)			0.792
AA	30 (39)	15 (47)	
AG	37 (49)	14 (44)	
GG	9 (12)	3 (9)	
TNFSF13B rs9514828, *n* (%)			0.793
CC	28 (37)	14 (44)	
CT	34 (45)	13 (41)	
TT	14 (18)	5 (16)	
TNFSF13B rs3759467, *n* (%)			0.257
TT	36 (47)	17 (53)	
CT	29 (38)	14 (44)	
CC	11 (14)	1 (3)	
Responder, *n* (%)	58 (76)	28 (88)	0.291

*Note*: *Italic* indicates that *p* < 0.05.

Abbreviations: AChR, acetylcholine receptor; AZA, azathioprine; CTX, cyclophosphamide; Fc γ receptor III‐A; FCGR2A, Fc γ receptor II‐A; FK506, tacrolimus; IVIg, intravenous immunoglobulin; MG, myasthenia gravis; MG‐ADL, MG‐related activities of daily living; MGFA, Myasthenia Gravis Foundation of America; MMF, mycophenolate mofetil; MTX, methotrexate; MuSK, muscle‐specific tyrosine kinase; PE, plasma exchange; QMG, quantitative myasthenia gravis; FCGR3A: RTX, rituximab.

### 3.2 Derivation and validation of the nomogram

**FIGURE 2:**
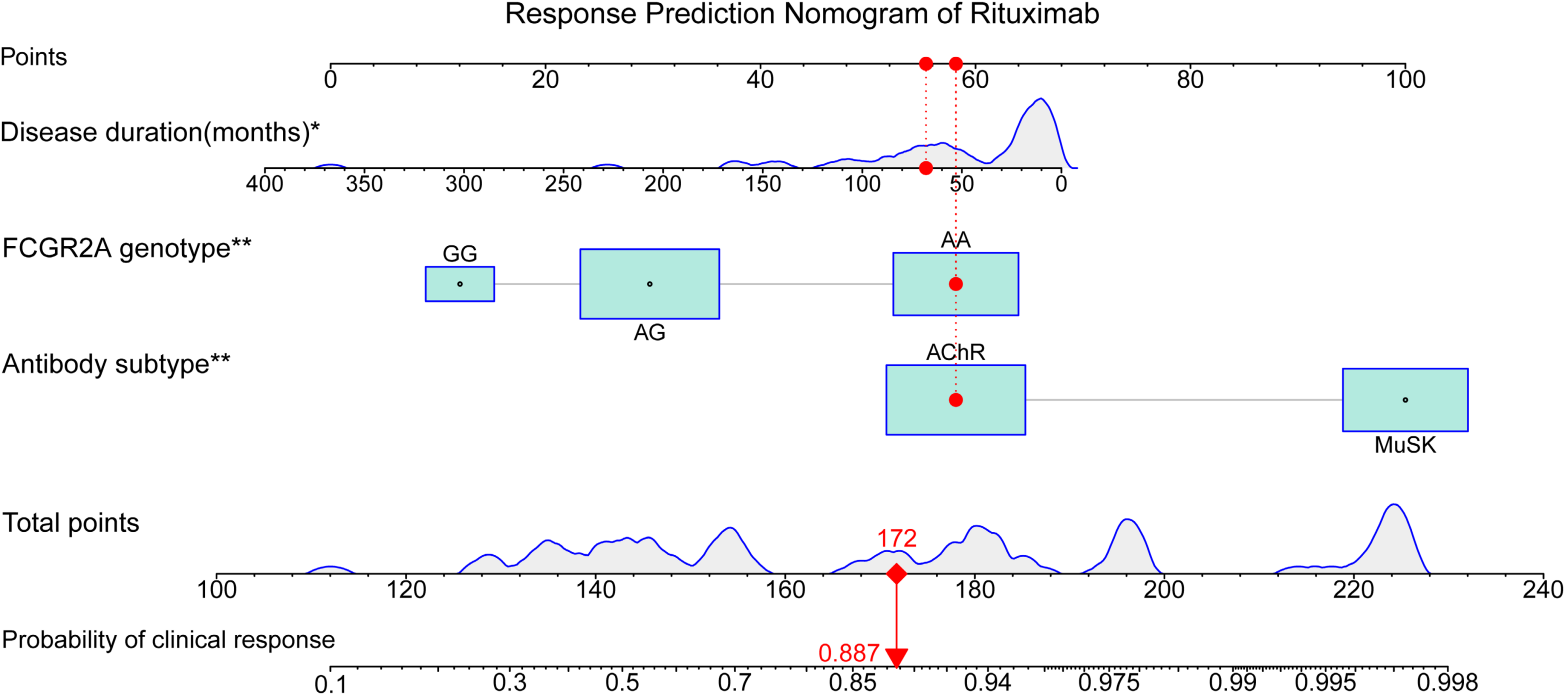
Nomogram to estimate the probability of clinical response in MG patients after low‐dose rituximab treatment. To use this nomogram, locate the position of each variable on the corresponding axis, draw a line from that point to the point axis to represent the number of points, add up the points from all of the variables, and draw a line from that total point value to the probability axis to determine the probability of clinical response at the lower line of the nomogram (as indicated by the red marker).

Univariate analysis revealed that several risk factors were significantly associated with clinical response. These factors include disease duration, antibody subtype, refractory property, QMG‐bulbar muscle score, ADL‐bulbar score, and *FCGR2A* rs1801274 genotype (*p* < 0.05) (Table 2). However, no significant association between *FCGR3A* (rs396991) or *TNFSF13B* (rs9514828, rs3759467) and the clinical response was identified in either a recessive model or dominant model (*p* > 0.05). We observed that the VIFs of these screened variables were 1.098, 2.458, 1.580, 2.723, 3.306, 1.135, and 4.216, respectively, demonstrating no multicollinearity among them. We did not consider the inclusion of the total QMG score in the model since the outcome is defined based on total QMG scores. Finally, we identified three predictive factors that were independently associated with the clinical response: disease duration with an odds ratio of (0.987 [0.975–0.999], *p* = 0.032), MuSK‐MG (19.8 [2.28–171], *p* = 0.007), AG (0.131 [0.023–0.762], *p* = 0.024), and GG (0.037 [0.003–0.450], *p* = 0.010) genotype in *FCGR2A* rs1801274 (Table 2). We then selected these three predictors to build a nomogram, which can calculate the total point for each post‐RTX MG patient and convert it to predicted probabilities of response (Figure 2).

**TABLE 2:** Univariate and multivariate logistic regression analysis for clinical response in the derivation cohort.

Variables	Subgroups	Univariate analysis (*n* = 76)			Multivariate analysis (*n* = 76)			
		OR	95% CI	*p* value	*β*	OR	95% CI	*p* value
Gender	Male	1						
	Female	2.094	0.598–7.328	0.247				
Age at onset		1.028	0.989–1.069	0.157				
Age at first RTX treatment		1.010	0.973–1.048	0.597				
Disease duration, months		0.987	0.977–0.998	*0.015*	−0.013	0.987	0.975–0.999	*0.032*
Antibody subtype	AChR	1				1		
	MuSK	9.846	2.072–46.781	*0.004*	2.983	19.8	2.28–171	*0.007*
MGFA classification	I	1						
	II‐III	2.867	0.169–48.74	0.466				
	IV‐V	7.000	0.302–162.202	0.225				
Other autoimmune diseases	NO	1						
	YES	0.819	0.225–2.978	0.762				
Thymoma	NO	1						
	YES	0.417	0.126–1.374	0.151				
Thymectomy	NO	1						
	YES	0.698	0.223–2.187	0.537				
Prednisone dosage, mg/d		0.989	0.956–1.023	0.518				
History of PE/IVIg	NO	1						
	YES	3.600	0.748–17.334	0.110				
Refractory MG	NO	1						
	YES	0.090	0.011–0.720	*0.023*				
QMG score		1.023	0.924–1.132	0.662				
Extraocular muscle score		1.076	0.838–1.381	0.565				
Bulbar muscle score		1.653	1.100–2.483	*0.016*				
Respiratory muscle score		1.389	0.784–2.462	0.260				
Gross motor score		0.925	0.807–1.060	0.262				
Axial motor score		0.943	0.518–1.717	0.848				
MG‐ADL score		1.060	0.913–1.230	0.444				
Bulbar score		1.560	1.087–2.237	*0.016*				
Respiratory function		0.936	0.512–1.713	0.830				
Limb score		0.883	0.639–1.219	0.448				
Ocular score		0.996	0.751–1.320	0.976				
FCGR3A rs396991	AA	1		0.982				
	CC	1.102	0.357–3.405					
	AC	0.964	0.165–5.649					
FCGR2A rs1801274	AA	1				1		
	AG	0.149	0.03–0.731	*0.019*	−2.032	0.131	0.023–0.762	*0.024*
	GG	0.089	0.013–0.625	*0.015*	−3.291	0.037	0.003–0.450	*0.010*
TNFSF13B rs9514828	CC	1		0.511				
	TT	1.052	0.308–3.589					
	CT	0.491	0.119–2.026					
TNFSF13B rs3759467	TT	1		0.792				
	CC	0.583	0.103–3.307					
	TC	0.778	0.139–4.352					

*Note*: *Italic* indicates that *p* < 0.05.

Abbreviations: AChR, acetylcholine receptor; FCGR2A, Fc γ receptor II‐A; FCGR3A, Fc γ receptor III‐A; IVIg, intravenous immunoglobulin; MG, myasthenia gravis; MG‐ADL, MG‐related activities of daily living; MGFA, Myasthenia Gravis Foundation of America; MuSK, muscle‐specific tyrosine kinase; PE, plasma exchange; QMG, quantitative myasthenia gravis; RTX, rituximab.

**FIGURE 3:**
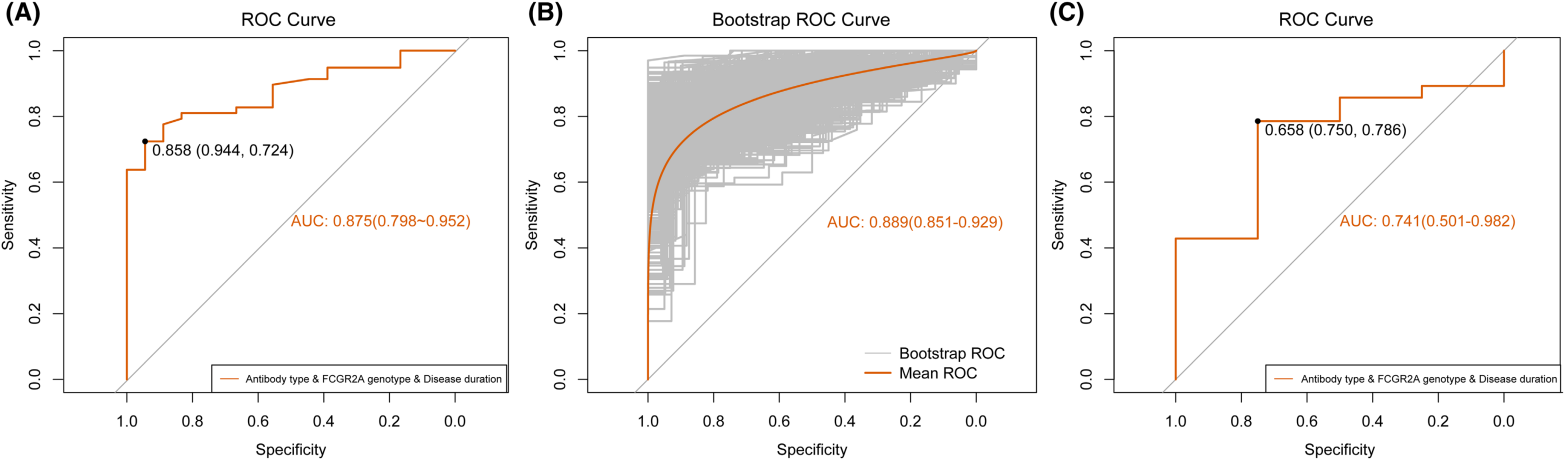
ROC‐AUC of nomogram. (A) Derivation cohort: ROC‐AUC = 0.875, 95% CI: 0.798–0.952. (B) Resampling: mean ROC‐AUC = 0.889, 95% CI: 0.851–0.929. (C) Validation cohort: ROC‐AUC = 0.741, 95% CI: 0.501–0.982. ROC‐AUC, Area under the receiver operating characteristic curve.

**FIGURE 4:**
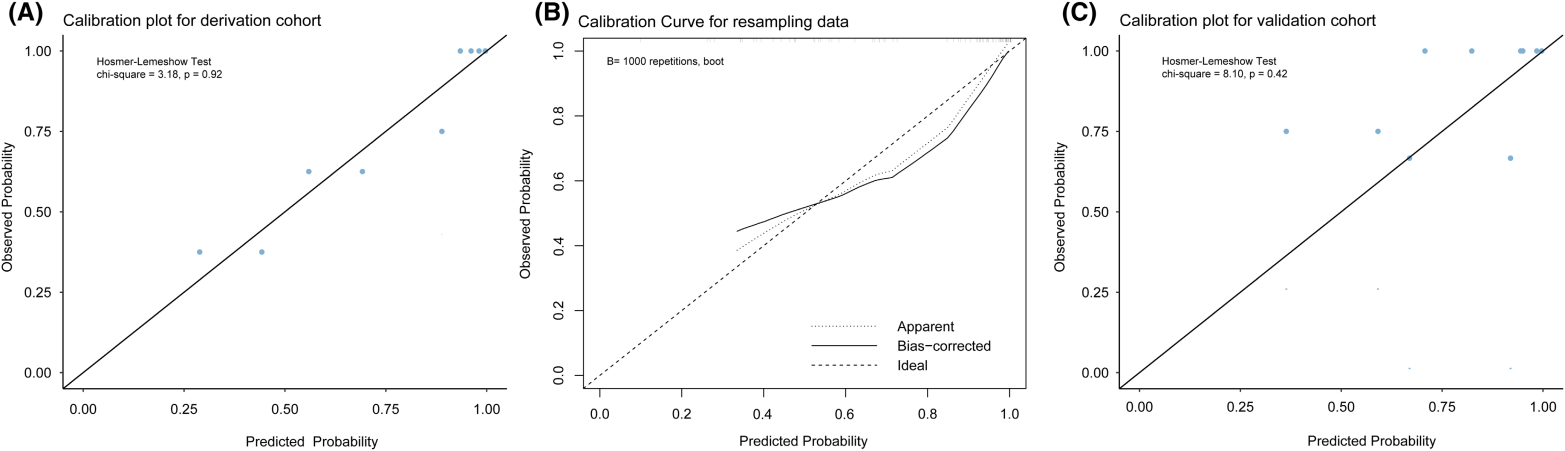
Model calibration of derivation cohort (A), resampling data using bootstrap (B), and validation cohort (C). The *x*‐ and *y*‐axes in the graph represent the predicted and actual response probabilities from the nomogram, respectively. The 45° gray line serves as the reference line, indicating perfect calibration power.

The model exhibited high discriminatory power with an AUC of 0.875 (95% CI: 0.798–0.952, Figure 3A) in the derivation cohort. The observed percentages of response corresponded well with the predicted possibilities (Hosmer–Lemeshow goodness‐of‐fit test: chi‐square = 3.181, *p* = 0.923, Figure 4A). Similar discriminatory power was exhibited in internal validation using resampling data (mean AUC = 0.889, 95% CI: 0.851–0.929, Figure 3B). On the calibration curve, the model's predicted probabilities were close to the observed probabilities (Figure 4B). The nomogram also performed well in the external validation cohort with good discrimination and calibration (AUC = 0.741, 95% CI: 0.501–0.982, Figure 3C; Hosmer–Lemeshow goodness‐of‐fit: Chi‐square = 8.098, *p* = 0.424, Figure 4C).

## 4 DISCUSSION

The effectiveness of RTX in treating MG has been confirmed in both randomized double‐blind controlled trials^15^ and real‐world studies.^16^, ^17^ However, not all patients in these studies achieved significant improvement. For both clinicians and patients, it is crucial to predict whether the effects of rituximab are clinically significant prior to making treatment‐related decisions. In our multicenter study, we identified that a shorter disease duration, a positive anti‐MuSK antibody, and the AA genotype in *FCGR2A* rs1801274 were significant predictors for achieving a clinical response within 6 months in MG patients treated with rituximab of 600 mg. Our predictive model might provide valuable information for the optimal use of rituximab in a real‐world setting by integrating these predictors into clinical practice.

We observed that a shorter duration from onset to rituximab exposure was a favorable predictor for clinical response. A prospective study also found that the clinical outcomes of rituximab were more favorable in new‐onset generalized myasthenia gravis compared with the refractory group.^18^ Consistent with our study, in the RINOMAX Randomized Clinical Trial,^15^ MG patients who experienced generalized symptoms within 12 months of onset showed a higher probability of minimal MG manifestations after receiving a single dose of 500 mg of rituximab. From a pathophysiological perspective, early initiation of effective immunotherapy appears to be more beneficial in achieving a clinical response by preventing structural damage to the neuromuscular junction. From an immunological standpoint, early immune responses are primarily mediated by plasmablasts and short‐lived plasma cells.^19^ With prolonged exposure, a selection process occurs under continuous antigen stimulation, promoting the development of long‐lived plasma cells and thereby maintaining humoral immunity.^20^, ^21^, ^22^, ^23^, ^24^, ^25^ It is plausible to speculate that early administration of RTX treatment by depleting immature, mature B cells, memory B cells, and plasmablasts may restrict the establishment of pathogenic antibody‐produced plasma cell pools.^26^

Compared with AChR‐MG, MuSK‐MG exhibits advantages in RTX therapy, as demonstrated in numerous clinical studies.^27^, ^28^ These differences may be attributed to the distinct pathogenesis of the antibodies: (1) Anti‐MuSK antibodies are primarily produced by short‐lived plasmablasts, whereas anti‐AChR antibodies are generated by long‐lived plasma cells and memory B cells.^29^, ^30^ Our previous studies have confirmed that low‐dose RTX can successfully delete B lymphocytes and reduce serum levels of pathogenic antibodies.^7^ The superior response of MuSK‐MG patients may be related to the shorter lifespan and faster exhaustion of pathogenic antibody‐producing cells (such as short‐lived plasmablasts). (2) Anti‐MuSK antibodies are predominantly of the IgG4 subtype, which generally does not activate complement, while anti‐AChR antibodies are of the IgG1 and IgG3 subtypes, which can amplify the damage by activating the complement cascade.^29^, ^30^ Treatment with anti‐CD20 antibodies cannot directly prevent the complement‐mediated membrane lysis and postsynaptic damage. (3) By binding to the postsynaptic membrane receptor, anti‐AChR antibodies can not only block signal transmission but also lead to endocytosis and therefore a loss of AChR densities. In contrast, anti‐MuSK antibodies predominantly disrupt the signaling pathways and impair the clustering of AChR receptors. The process of structural repair may require more time compared to the adjustment of functional dysfunction. (4) Anti‐AChR antibodies can interfere with the muscle regeneration process by regulating myogenic markers, while the effect of anti‐MuSK antibodies on regeneration remains unclear.^31^

Studies have also demonstrated that genetic factors, specifically SNPs, are associated with interindividual variations in response to RTX among patients with autoimmune diseases. For instance, Caucasian rheumatoid arthritis (RA) patients with the AA genotype in *FCGR2A* rs1801274 exhibited a higher remission rate 6 months after RTX treatment.^14^ Similarly, RA patients displayed a better response when possessing the *FCGR3A* rs396991‐V158 allele.^32^, ^33^ Consistent with previous researches, our study also identified that *FCGR2A* rs1801274‐G was an unfavorable factor for RTX response in an additive model. However, we did not observe the association of *FCGR3A* rs396991‐A with the clinical response. In vitro investigations have shown that rituximab can delete IgG‐opsonized B cells by complement‐dependent cytotoxicity (CDC), antibody‐dependent cell‐mediated cytotoxicity (ADCC), and antibody‐dependent phagocytosis (ADCP), which require recognition of bound IgG on opsonized target cells by the Fcγ receptor (FcγR) expressed by effector cells, such as monocytes, macrophages, neutrophils and natural killer cells.^34^, ^35^, ^36^ Interestingly, FcγRIIA exhibits a higher affinity for IgG1 than that of FcγRIIIA.^37^ Polymorphisms within the genetic coding regions of these two receptors could also affect their affinity with the Fc fragment of rituximab, thereby showing an impact on depleting B cells.^38^, ^39^ FcγRIIA carrying His (*FCGR2A* rs1801274‐A) rather than Arg at position 131 showed a higher affinity for IgG1.^40^, ^41^, ^42^ Similarly, the V158 variant in *FCGR3A* (rs396991‐C) demonstrated a greater affinity for IgG1.^43^, ^44^ Therefore, polymorphisms in Fcγ receptors could potentially impact the therapeutic efficacy of RTX by either augmenting or diminishing ADCC or ADCP mediated by Fcγ receptors carried by effector cells.

Our study had several limitations: First, it was a retrospective study with a relatively small sample size. Second, incomplete medical records and unavoidable missing data led to the exclusion of certain variables, including the initial dosage of steroids and the frequency of exacerbations before RTX treatment. Third, since there was heterogeneity in response between the external and the internal cohort, the findings from this study require further validation in larger and multiple cohorts to establish their generalizability and reproducibility. Lastly, future research is imperative to identify predictors of achieving minimal MG manifestations and the likelihood of disease relapse in MG patients undergoing RTX treatment.

In conclusion, we have developed and validated a nomogram for predicting the probability of clinical response to rituximab based on baseline clinical and genetic characteristics. The nomogram holds potential utility in guiding clinical decision‐making for MG patients in low‐dose RTX treatment.

## AUTHOR CONTRIBUTIONS

Jianying Xi and Ting Chang designed the study, revised, and approved the manuscript. Chongbo Zhao supervised the study. Yufan Zhou and Rongjing Guo analyzed the clinical data, conducted statistical analysis, and drafted the manuscript. Yufan Zhou, Xingyu Xia, Sisi Jing, Jun Lu, Zhe Ruan, Sushan Luo, and Xiao Huan reviewed and collected clinical and laboratory data.

## FUNDING INFORMATION

This research was supported by the National Natural Science Foundation of China (No.82071410, No.8210052671, and No.82001335), the National Key Research and Development Plan of China (2022YFC3501304), and the Shanghai Municipal Science and Technology Major Project (No.2018SHZDZX01), and ZJ Lab.

## CONFLICT OF INTEREST STATEMENT

The authors declare that the research was conducted in the absence of any commercial or financial relationships that could be construed as a potential conflict of interest.

## Data Availability

The data that support the findings of this study are available on request from the corresponding author. The data are not publicly available due to privacy or ethical restrictions.
